# Galvanic microcells as control agent of indoor microorganisms

**DOI:** 10.1038/srep35847

**Published:** 2016-10-27

**Authors:** Wojciech Spisak, Andrzej Chlebicki, Mariusz Kaszczyszyn

**Affiliations:** 1 Research & Development Centre, Alcor Ltd.,Kępska 12, 45-130 Opole, Poland; 2 W. Szafer Institute of Botany, Polish Academy of Sciences, Lubicz 46, 31-512 Kraków, Poland

## Abstract

Today, fungicides are part of the basic tool kit for indoor surface maintenance. However, fungi develop resistance to fungicides, which consequently accelerates the evolution of virulence. Fungicides also carry the risk of adverse effects in humans. Galvanic microcells are a new tool for fungal control on indoor surfaces. We used two types of electrodes, Zn and Cu, with two potential anti-fungal mechanisms: the oligodynamic action of the metal ions themselves and the electricidal effect of the current between the electrodes. The size of the inhibition zone is related to the distance between the electrodes. We hypothesized that the unique geometric properties of the observed inhibition zone could be modelled using multi foci curve Cassini ovals. Moreover, the size of the inhibition zone possessed two maximum values, while the shape of the observed inhibition zones correlated with the shape of the electric field strength. The control activity of the galvanic microcells correlated with decreasing water content in building materials. Thus, this acute antifungal system works the best in damp building environments where the risk of fungal contamination is highest.

The long history of studies of human activity shows that in changing the world, we have changed ourselves to the point that spending large amounts of time indoors is now a fundamental characteristic of the human species^1^. The 2001 National Human Activity Pattern Survey estimated that people in developed countries spend nearly 90% of their time indoors and that time spent indoors has remained fairly uniform over the past few decades^2^. Humans share indoor spaces with diverse populations of microorganisms from all three domains that create bioaerosols^3^ and colonize indoor surfaces. Fungal contamination of indoor ecosystems has always been of interest in connection to building structure biodeterioration and serious effects on human health^4^. Risk assessments for human exposure to indoor fungal bioaerosols and mycotoxins indicate that these factors are a major contributor to sick building syndrome^5^. Fungal contamination in buildings is very often connected with environmental disasters, a notable example being Hurricane Katrina, as reported by Bennett in “The fungi that ate my house“^6^. The climate crisis has also revealed a secondary, more insidious and more pervasive crisis, namely, the biodiversity crisis. Humans have reduced the abundance of numerous species in what has come to be known as the “sixth mass extinction“^7^. Multiple efforts should be implemented to stop this trend, and one possible step is to reduce the use of biocidal chemicals. Unfortunately, fungicides are very important not only for agriculture but also for the basic maintenance of indoor surfaces. One risk posed by these substances is the development of fungal resistance and the accelerated evolution of virulence in pathogenic fungi connected with human activities^8^. Fungal resistance occurs through similar mechanisms as in bacteria, including the degradation and inactivation of biocides (antibiotics), decreased membrane permeability, the use of repair mechanisms, and phenotypic modulation^9^. Fungicides may also have adverse effects in humans.

We propose galvanic microcells as a new tool for fungal biocontrol on indoor surfaces. Specifically, we hypothesize that indoor surfaces can be protected against initial pathogenic fungal colonization through the formation of an electrochemically generated, ionic conductive environment using galvanic microcells. Electrochemical galvanic activity in building materials can occur only in the presence of water-based electrolytes freely bound by adsorption and absorption forces in capillary spaces or in water freely available on the surface. Moreover, humidity is a key factor in the initiation of microbial contamination in buildings. The availability of moisture for microbial life in porous materials can be described by the water activity parameter, which is defined as the ratio of the vapour pressure of water in a material to the vapour pressure of pure water at the same temperature. Water activities from 0.6–0.7 are the lowest values necessary to initiate fungal growth on materials containing enough nutritive substances^10^. Fungal activity, as well as the ability to colonize new surfaces, increases as the water activity approaches 1, i.e., when water is freely available, which also corresponds to when galvanic activity should be maximal. The transient activity of galvanic microcells is a key difference compared with the permanent activity of classical fungicides. The control activity of galvanic microcells correlates with the water content in building materials, and galvanic microcells can turn to rust when there is an environmental risk of uncontrolled indoor dampness or decay. Certain metals and their salts are known to exhibit considerable biocidal properties. The ability of very small amounts of several metal ions to inhibit or kill microorganisms, known as the oligodynamic effect, was first discovered in 1893 by von Nageli^11^. Several metal ions, especially heavy metals, show this effect to varying degrees, the most well-known examples being silver and copper.

## Results

**Figure 1:**
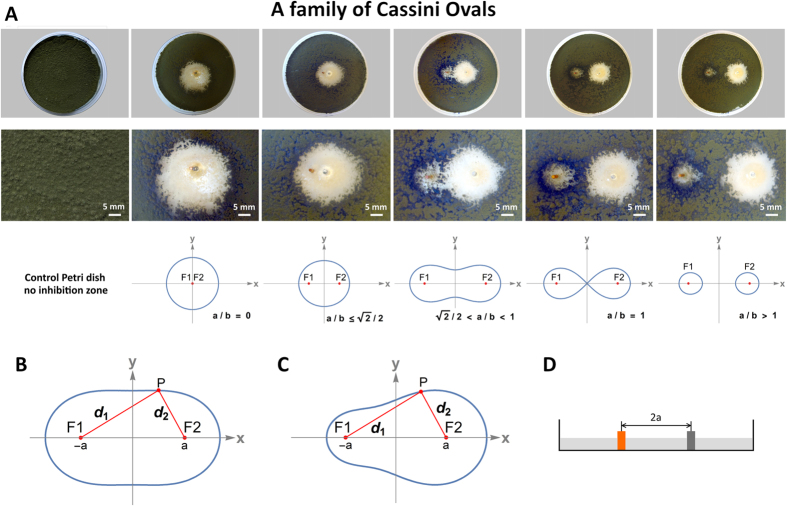
Inhibition zones (IZs) of copper-zinc galvanic cells for *Cladosporium cladosporoides*. (**A**) Proposed correlation of IZ overhead views with the shapes of Cassini ovals; (**B**) A Cassini oval with foci F1 and F2 on the x-axis defined by the equation d_1_d_2_ = b^2^; (**C**) A disturbed Cassini oval defined by the equation d_1_^α^d_2_^β^ = b^2^; (**D**) Schematic of a Petri dish with metal bar zinc and copper electrodes used in the experiments.

Oligodynamic metals can be arranged in pairs to form galvanic cells when immersed in an ion-conducting electrolyte. The systems we examined had electrode dimensions and separation distances on the scale of micrometers and millimetres and were thus called microcells. We predict that galvanic microcells offer two potential anti-fungal mechanisms: the oligodynamic action of the metal ions and the “electricidal effect” of the current between the electrodes. The oligodynamic actions of a very small concentration of positively charged metal ions have been studied most extensively for copper and silver, although the antimicrobial properties of other metals and alloys have also been investigated^12^. The ability of the “electricidal effect” to reduce bacterial biofilm formation on solid surfaces has been already demonstrated by Pozo *et al*. in experiments involving prolonged exposure to low-intensity (e.g., microampere) direct current^13^. We have named the visible influence of galvanic current on fungal growth Galvanic Protection Action (GPA). In our experiments, we used nine different metals (gold, silver, copper, titanium, iron, aluminium, bismuth, zinc, and magnesium) with a wide range of ion bioactivities and electrochemical standard potentials from −2.70 V for magnesium to +1.83 V for gold. Initial experiments were performed on a millimetre scale using electrodes in the form of 1.2-mm diameter, 7-mmlong bars fixed in the middle of Petri dishes (Fig. 1D). We tested the antifungal activity of the metals alone, as well as 45 types of galvanic cells with different metal electrode combinations separated by 4.2 mm between the electrode centres. The strongest antifungal activity was observed for copper-zinc cells, which were therefore examined in more detail. This observation agreed with previous results from clinical applications of mixtures of copper and zinc salts in pyogenic skin infections and traumatic wound treatments^14^. The basic preparation of copper and zinc salts for medical applications is known as Dalibour’s Water, named for Jacques Dalibour, the French surgeon in the Army of Louis XIV of France^15^. Copper, zinc and their alloys have well-known antimicrobial properties and are still often used today.

In our experiments, we used very common indoor fungi such as *Cladosporium cladosporioides* and *Ulocladium atrum* isolated from the indoor spaces of old flats in Cracow. Because natural stones are among the oldest and most stable construction materials, we also used rock-inhabiting fungi (RIF) that are recognized as the most important endoliths. Moreover, natural stone materials are increasingly used in contemporary architecture. Concrete, lime plasters and paints can also be deteriorated by RIF. Among RIF, we chose *Penicillium spinulosum* and black yeast (BY) that resist chemical attack and anti-microbial treatments^16^,^17^. We used BY such as *Aureobasidium pullulans*, as well as yeast *Rhodotorula mucilaginosa* that is often identified in settled dust samples from the ducts of air-conditioning systems^18^. Strains of these fungi were prepared according to standard microbiological techniques^19^. Control activities (i.e., GPA) towards the different fungi were determined by measuring the inhibition zone in disk diffusion susceptibility tests. Interpretation of the tests was problematic for galvanic cells because of the unusual forms of these zones. The size of the zone of inhibition is usually related to the diameter or radius of the clear zone, which forms a more or less regular disk around the test product. However, we observed disk-shaped inhibition zones only when the different metals were tested alone. For the galvanic cells, increasing electrode distances caused the inhibition zones to change from disks to more or less deformed ellipses to peanut-shaped ovals that finally split into two disjointed ovals. We hypothesized that the unique geometric properties of the observed inhibition zones can be modelled using multi foci curve Cassini ovals (Fig. 1A).

Cassini ovals are a family of quartic curves described by a point P, such that the product of the distances from two fixed points separated by distance **2a** equals the constant **b**^**2**^ (Fig. 1B). In our case, the fixed points are the centres of the electrodes, which are separated by a distance of **2a**. The shape of the Cassini curve depends on the parameters **a** and **b** and is generally categorized by evaluating the ratio **a/b** (Fig. 1A). If **a** < **b**, the curve is a single loop with an oval or dog bone shape. The case **a** = **b** produces a Lemniscate of Bernoulli. If **a** > **b**, then the curve consists of two loops. In our experiments, all of these shapes were observed. If we locate the origin (0,0) of the Cartesian plane at the midpoint of the two foci **F1** and **F2** and choose the x-axis as the line joining them, then the curves will be symmetric with respect to both axis and origin. In our experiments, we observed that the margins of the inhibition zones were symmetric with respect to the x-axis and were strongly asymmetric with respect to the y-axis and the origin. The Cassini ovals are described by the quadratic polynomial Cartesian equation:


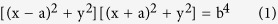


In two-centre bipolar coordinates, the Cassini ovals are defined by the equation:


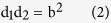


where d_1_ and d_2_ denote the ranges F1 to P and F2, respectively (Fig. 1B).

**Figure 2:**
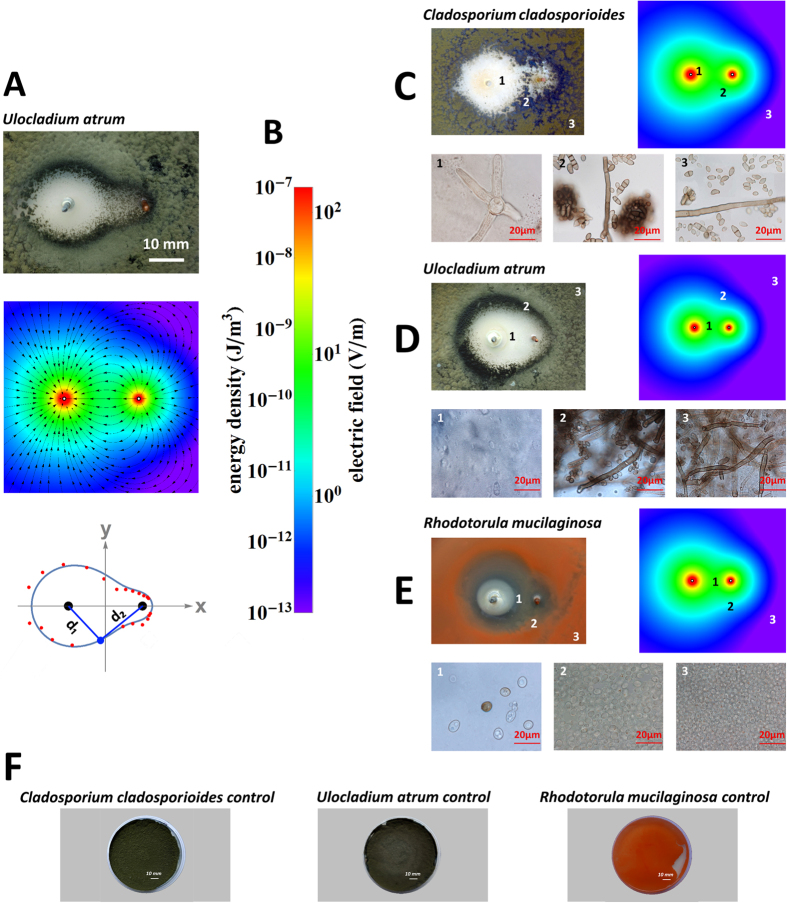
Correlation of the observed inhibition zones with the geometry of the electric field generated by zinc-copper galvanic cells. (**A**) IZ of *Ulocladium atrum*, electric field strength and disturbed Cassini oval equation d_1_^1.49312^d_2_^0.50688^ = 14.43225^2^ calculated from experimentally measured points from the IZ margin; (**B**) Colours show the energy density and electric field; (**C**) *Cladosporium cladosporioides*, (**D**) *Ulocladium atrum*, (**E**) *Rhodotorula mucilginosa* IZ shape and corresponding electric field generated by galvanic cell copper-zinc with micrographs of mycelium.

We hypothesized that the asymmetric curves of the inhibition zones observed in our experiments can be represented by the equation:


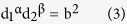


where the exponents alpha and beta are correlated with the antifungal activity of the cathode and anode (Fig. 1C). The exponents **α** and **β** were incorporated into the classical Cassini oval equations using numerical methods based on experimental measurements. For each plate, the positions of 20 points along the inhibition zone boundary were determined by a trained observer, and the distances d1 and d2 were measured from these points. The values of **α** and **β** were then calculated based on the measured distances(see Methods). As a result, we obtained equations for curves that cover the margins of the observed inhibition zones (Fig. 2A),which we used to calculate the areas of the inhibition zones. The area of the zone of inhibition is usually related to the level of antimicrobial activity. Because the inhibition zones for the galvanic cells corresponded to Cassini ovals,we can expect two maximum values for areas clear of fungi. The first maximum value, for **a/b** = 0, corresponds to galvanic microcells whose electrodes touch. The second local maximum value, for **a/b** = 1, corresponds to electrode distances that results in inhibition zones in the form of lemniscates. The electrode distance in the second maximum appears to be the best configuration for antifungal performance and cost efficiency in galvanic surface protection systems. The shapes of the observed inhibition zones are correlated with the shape of the electric field (Fig. 2A–D). The electric field was visualized using finite element modelling under idealized vacuum conditions and spherical electrodes with the same diameters and at the same distances as the real metal bar electrodes (see Methods).

**Figure 3:**
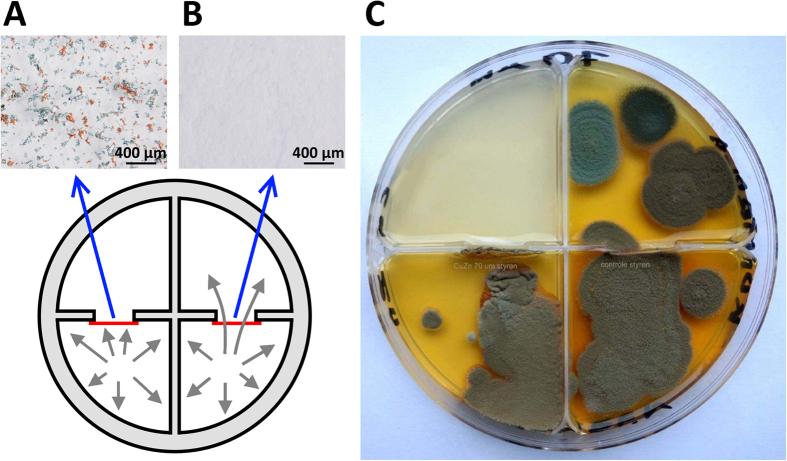
Barrier protection by copper-zinc galvanic microcells against fungal colonization (*Penicillium spinulosum*). (**A**) styrene blister with copper-zinc microcells; (**B**) pure styrene blister of the same size and thickness; (**C**) Petri plate showing the inhibition effect of the zinc-copper galvanic microcells.

For the practical application of GPA for indoor surface protection, the size of the galvanic cells should be in the range of micrometers. Thin transparent polystyrene blisters with a 70-μm thickness were used as a barrier against fungal proliferation to demonstrate the efficacy of GPA using microelectrodes in the form of metal grains with sizes below 90 μm (Fig. 3A). Blisters 9 × 15 mm in size were cut from polystyrene film formed from a polystyrene solution in toluene and powdered by zinc and copper electrodes before drying. Due to the deposition method, the distribution of the electrodes was random (Fig. 3A). A vertically oriented styrene blister with zinc-copper microcells was fixed in a window cut between two segments of four-chamber Petri dishes (see Supplementary Information). Between the next two segments of the same Petri dish, a pure styrene blister of the same size and thickness (70 μm) was fixed as a control (Fig. 3B). Even after 36 days, fungal mycelia of *Penicillium spinulosum* were unable to across the blister containing GPA, whereas a pure styrene blister of the same thickness was not a barrier to fungal proliferation (Fig. 3C).

All fungi produce bigger (more swollen) conidia and hyphae in areas near electrodes. Fungi that generate biofilms react differently on GPA compared with hyphomycetous species.The inhibition zone for biofilm-producing fungi is smaller and includes mostly living conidia that can proliferate, whereas hyphomycetous fungi are more sensitive to GPA and create bigger inhibition zones that include mostly dead conidia or strongly swollen hyphae that are distinctly pale. Both groups of fungi induce the production of melanin in the conidia and hyphae of mycelium surrounding the margin of the inhibition zone and the zinc electrode. Gadd^20^ noted that the presence of copper in solid medium can induce the production of melanin-pigmented hyphae in *Aureobasidium pullulans*. The control activity of galvanic microcells enables the reduction of fungal colonization in damp environments, where the risk of fungal contamination is highest. The inhibition zones for galvanic cells are disturbed Cassini ovals with two maximum-value areas clear of fungi. This strong systems free from organic antifungal chemicals and can be used in many indoor areas. Oligodynamic and electricidal effects may act additively in GPA.

## Methods

### Cassini ovals

Numerical determination of the exponents α and β to search for the best approximation of the average coefficient b.

The Cassini ovals are described by the quadratic polynomial Cartesian equation:


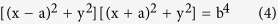


This method uses a Cassini oval equation in two-centre bipolar coordinates, which for a normal oval is:


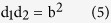


and for a distorted oval is:


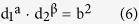


where d1 and d2 denote the distances to centres of the anode and cathode. For each plate, the positions of 20 points along the inhibition zone boundary were determined by a trained observer, and the distances d1 and d2 for each point were measured with 1-mm accuracy. The algorithm performs n-steps, counting the value of b for each measurement point. The values of α and β are determined at each step, taking into account the ratio between them.


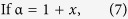



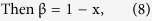


meaning that at each step, α increases by the same amount that β decreases (setting a constant growth value of x = 0.01). α ∈ (1.2) and β ∈ (0.1). After determining α and β for a given step, the algorithm computes the b value of the model for all pairs of d_1_, d_2_, corresponding to the points on the circumference of the oval. The program keeps track of the deviation of b by setting b_min_ and b_max_for the step value. The result is a pair of α and β for which the calculated b for all points reaches the smallest dispersion (i.e., the lowest value for b_max_ − b_min_). The curves of Cassini ovals were subsequently plotted in Wolfram Mathematica R 10 https://www.wolframalpha.com version PRO based on the calculated values of α, β, and b and from the known value of factor a, defined as half the measured distance between the electrode centres.

### Measurement of electrode potential and cell voltage

The electric potential of the cathode and anode and the cell voltage between the metal bar electrodes were measured with a True-rms Digital Multimeter model 289 (Fluke Corporation, USA).

A silver chloride electrode Ag/AgCl/Sat. KCl with the standard hydrogen electrode potential +0.197 V (Hydromet, Gliwice/Poland) was used as a reference electrode.

### Finite element modelling (FEM) of the electric field

The FEM package used to create the electric field models was Wolfram Mathematica R10 multiphysics https://www.wolframalpha.com. The standard electrode potential was −0.7628 V for zinc and +0.337 V for copper relative to the standard hydrogen electrode and spherically shaped electrodes were used for the geometric electric field calculations.

### Electrodes for galvanic cells

Galvanic cells for experiments on the millimetre scale were constructed with electrodes in the form of 1.2-mm diameter, 7-mm long bars fixed in the middle of polystyrene Petri dishes. Special high-grade zinc with a minimum zinc content of 99.5% was used for the anodes, and Electro Tough Pitch Copper with a minimum copper content of 99.5% was used for the cathodes. Electrodes for experiments on the micrometre scale were in the form of metal grains from metallic powders deposited randomly on 70-μm-thick polystyrene blisters. Zinc powder(Libra Corp., Trzebinia/Poland) ground from solid metal with a minimum 99.5% zinc content was produced by mechanical comminution in a ball mill. Zinc powder particles have metal strip shapes and diameters below 90 μm. Electrolytic copper powder with a minimum copper content of 99.8% (Libra Corp., Trzebinia/Poland) was generated from cathodic precipitation via the electrolysis of aqueous copper sulphate. The powder particles have a dendritic shape with a diameter below 63 μm.

### Fungal isolation

Fungal isolation following fungal species were used: *Aureobasidium pullulans* var. *pullulans*, *Cladosporium cladosporoides*, *Penicillium spinulosum*, *Rhodotorula mucilaginosa*, *Talaromyces diversus*, *Ulocladium atrum* and *Wallemia mellicola*. Fungi were collected using an MAS-100 Eco air sampler. Standard Petri plates (90-mm diam.) containing PDA medium were used. The resulting fungal colonies were moved to fresh PDA medium and cultivated at room temperature under dark/light conditions. Our molecular analysis of the Internal Transcribed Spacer (ITS1-5.8S-ITS2) identified the yeast as a *Rhodotorula mucilaginosa* strain with 98% identity to the sequence of *R. mucilaginosa*, GenBank accession number EU285542.1 (see subchapter ‘Molecular analysis’ Supplementary Information).

### Growth observations

Observations were made after 2, 3, 7, 10, 14, and 30 days of growth. For microscopic observation and photography of fungal morphological structures, Nikon SMZ 1500 and Nikon Eclipse 80i light microscopes were used. Micrographs were obtained using a Nikon DS-Ri1 Nikon camera.

### Experiments

Fungal mycelium with spores were removed to 100-ml beakers containing 20 ml of 0.9% agar and stirred with a magnetic stirrer approximately every 30 sec. A solution with suspended conidia was smeared on the medium surface (PDA medium, pH 5.6) in Petri plates containing metal electrodes using a Drigalski spatula approximately 10 mm long and 1 mm diam. The cultures were then maintained at room temperature in the dark. After four days, the diameter of the growing halo was measured. Plates were documented photographically using a Cyber-shot DSC-RX 100 camera. In the subsequent experiments, a solution with suspended conidia was placed in a diffuser and sprayed on to the medium surface. Experiments with electrodes were performed on sets of 38 dishes with different distances between electrodes (Supplementary Information – see Figure).

## Additional Information

**How to cite this article**: Spisak, W. *et al*. Galvanic microcells as control agent of indoor microorganisms. *Sci. Rep*. **6**, 35847; doi: 10.1038/srep35847 (2016).

**Publisher’s note:** Springer Nature remains neutral with regard to jurisdictional claims in published maps and institutional affiliations.

## Supplementary Material

Supplementary Information
