# Supplementary material for: Galvanic microcells as control agent of indoor microorganisms
Source: Sci Rep. 2016 Oct 27;6:35847. doi: 10.1038/srep35847 (PMC5081506; doi:10.1038/srep35847)
Supplement: Supplementary Information [file srep35847-s1.pdf]

Galvanic microcells as control agent of indoor microorganisms

Wojciech Spisak[1], Andrzej Chlebicki[2*]& Mariusz Kaszczyszyn[1]

Supplementary Information

METHODS

Molecular analysis

The Internal Transcribed Spacer (ITS1-5.8S-ITS2) of nuclear ribosomal DNA (nrDNA) was amplified and sequenced from the culture. The mycelium of *Rhodotorula mucilaginosa* was scraped off the medium into test tubes with CTAB buffer and glass bulbs. The probes were disrupted and later incubated for 10 minutes at 65°C. Chloroform/phenol (Roth) was added, and the solution was centrifuged at 14000 rpm. The supernatant was treated with 2 vol. of ice-cold ethanol (70%). The samples were stored overnight at -20°C for DNA precipitation and were then centrifuged for 5 minutes at 14000 rpm. The resulting pellet was washed in ethanol, dried and dissolved in ddH_2O.

Amplification of the ITS1-5.8S-ITS2 region was accomplished using a touchdown polymerase chain reaction (PCR). Amplification was performed in a 25-µl reaction volume in an *Eppendorf Mastercycler Nexus* thermocycler containing 1x REDTaq PCR reaction buffer (Sigma-Aldrich), 0.25 mmol dNTPs in an equimolar ratio, 0,01 mg/ml bovine serum albumin, 0.05 unit/µl U Taq DNA polymerase (Sigma-Aldrich) and 0.2 µmol each of the ITS1-F and ITS4 primers. Stock DNA (1 µl) was added to each reaction as a template. The PCR protocol was as follows: 3 min 94°C, 10 cycles (30 sec 94°C, 30 sec 60°C → the annealing temperature is reduced by 1°C every successive cycle, 1 min 72°C), 25 cycles (30 sec 94°C, 30 sec 50°C, 1 min 72°C), and a final 7-min extension at 72°C was performed, after which the reaction was held at 4°C until further processing. PCR products were screened on 1% agarose gel, then purified using ExoSAP-IT PCR Product Cleanup (Affymetrix). Sequencing was completed using a BigDye Terminator v3.1 Cycle Sequencing Kit (ThermoFisher Scientific) together with BDX64 Sequencing Enhancement Buffer (Nimagen), according to the manufacturer's protocol (Nimagen) for 32x dilution. The sequencing reaction protocol was as follows: 3 min 96°C, 30 cycles (10 sec 96°C, 5 sec 50°C, 2 min 60°C), and it was performed in an *Eppendorf Mastercycler Nexus* thermocycler. The products were sequenced with the same pair of primers used for amplification. To remove unincorporated reaction components, the resulting products were cleaned using ethanol/EDTA precipitation. Labelled fragments were separated on POP-7 polymer, using an ABI Prism 3130 automated DNA sequencer.

Sequences were aligned and adjusted using ClustalX2.1. The phylogenetic tree was constructed with the Molecular Evolutionary Genetics Analysis (MEGA) software version 6.06 (Tamura et al. 2013).

Our search identified the yeast as *Rhodotorula mucilaginosa* strain LSU1ach4.F with 98% identity to the sequence of *R. mucilaginosa*, GenBank accession number EU285542.1.

Experiments

Experiments with electrodes in the form of 1.2-mm-diameter, 7-mm-long metal bars fixed in the middle of Petri polystyrene dishes were performed for each fungus on sets of 38 dishes with different distances between electrodes. The gap L (see Figure) between electrodes ranged from 0 mm (electrodes in touch) to 30 mm. Example results for *Cladosporium cladosporioides* with control dishes are shown in Figure.

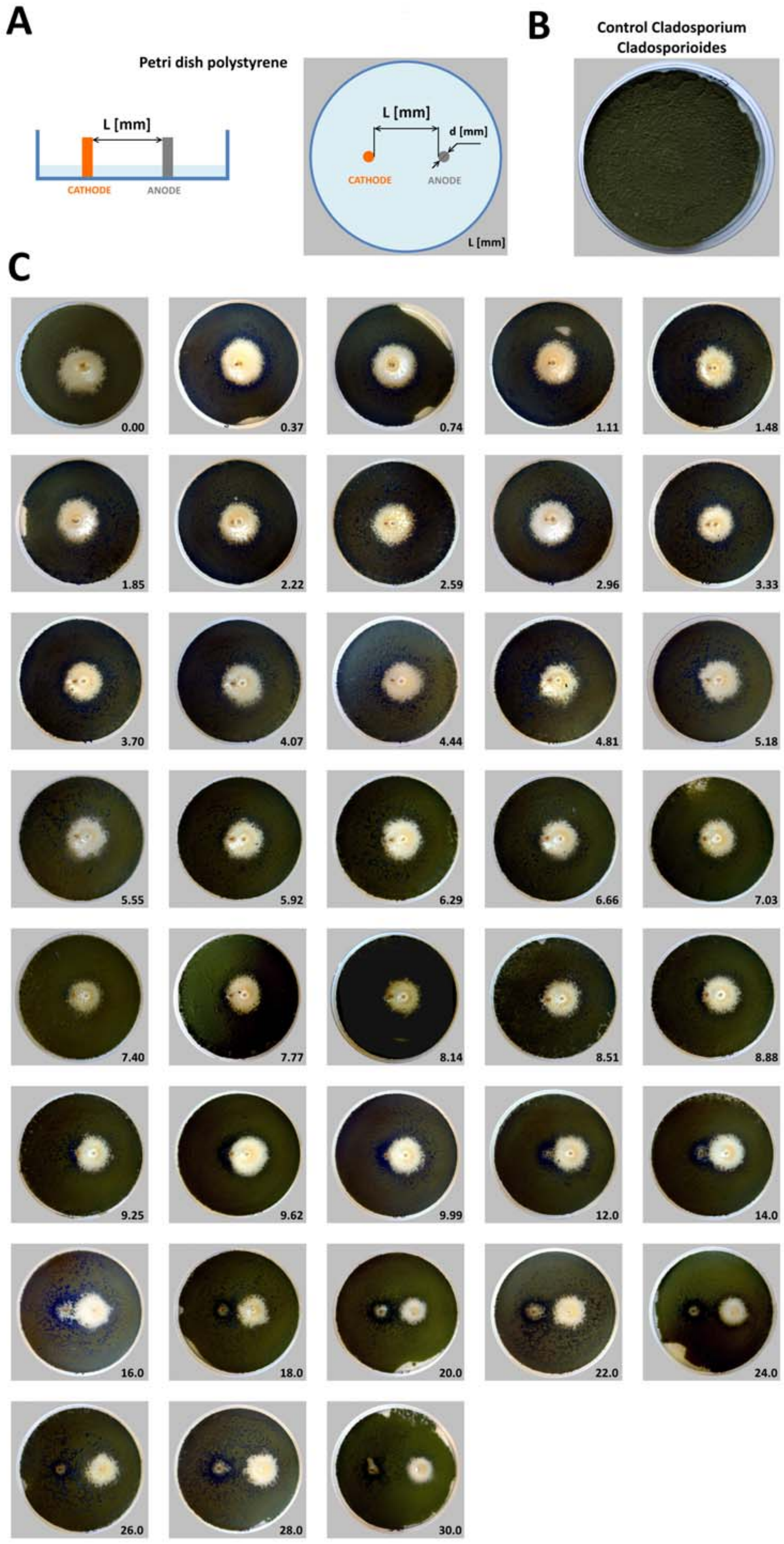
A
Petri dish polystyrene
L [mm]
CATHODE
ANODE
L [mm]
d [mm]
CATHODE
ANODE
L [mm]
B
Control Cladosporium
Cladosporioides
C
0.00
0.37
0.74
1.11
1.48
1.85
2.22
2.59
2.96
3.33
3.70
4.07
4.44
4.81
5.18
5.55
5.92
6.29
6.66
7.03
7.40
7.77
8.14
8.51
8.88
9.25
9.62
9.99
12.0
14.0
16.0
18.0
20.0
22.0
24.0
26.0
28.0
30.0

Figure. Correlation of observed inhibition zones with gap L between electrodes for *Cladosporium cladosporioides*. (A) - arrangement of electrodes; (B) - control; (C) - full ranges of examined distances. The value of “L” is placed in the lower-right corner of the photo samples.

In the next experiment, vertically oriented styrene blisters with zinc-copper microcells and a pure styrene blister as a control were separately dipped in PDA medium in four-chamber Petri dishes. We used the strain *Penicillium spinulosum* isolated from deer horn (Chlebicki & Spisak 2016). Petri dishes were stored at room temperature in light/dark conditions and studied after 36 days of incubation.

Chlebicki, A., Spisak, W. *Amauroascus kuehnii* and other fungi isolated from deer horn in Poland. *Polish Botanical Journal* **61**(1), 161-166 (2016).

Tamura, K., Stecher, G., Peterson, D., Filipski, A., Kumar, S. MEGA6: molecular evolutionary genetics analysis version 6.0. *Mol Biol Evol.* **30**, 2725–2729 (2013).
